# Generalist vs. specialist strategy shapes microbiomes in blood feeding parasite *Polyplax serrata*

**DOI:** 10.3389/fmicb.2025.1720127

**Published:** 2025-11-21

**Authors:** Damián Dedecius, Jakub Kolář, Jana Martinů, Jan Štefka, Eva Nováková, Václav Hypša

**Affiliations:** 1 Department of Parasitology, Faculty of Science, University of South Bohemia, České Budějovice, Czechia; 2 Institute of Parasitology, Biology Centre, ASCR, v.v.i, .České Budějovice, Czechia

**Keywords:** microbiome, beta diversity, lice, generalist and specialist, insect symbionts

## Abstract

Insects live in association with bacterial communities, collectively referred to as the microbiome. Microbiome composition varies widely across insect taxa and is shaped by multiple factors, including host phylogeny, environmental conditions, geographic distribution, and nutritional ecology. One hypothesis is that microbiome composition may also reflect whether the host adopts a generalist or specialist ecological strategy. We tested this hypothesis using the sucking louse *Polyplax serrata*, which offers several advantages as a model system. First, as permanent ectoparasites, lice inhabit a relatively stable and simplified environment, thereby minimizing potential confounding variables. Second, within *P. serrata*, two closely related lineages have been identified: one restricted to a single rodent host (*Apodemus flavicollis*), and the other exploiting two hosts (*A. flavicollis* and *A. sylvaticus*). We analyzed and compared microbiome structure in these two lineages using 16S rRNA gene amplicon sequencing. While alpha diversity did not differ between the lineages, beta diversity differed significantly, particularly in pairwise dissimilarities among individual samples. These results suggest that in *P. serrata*, host specialization strategy influences microbiome diversity, with the “generalist” lineage harboring more heterogeneous communities. This finding extends previous observations on ecological divergence between the two lineages, showing that closely related cryptic species with highly similar genomes, living sympatrically in the same environment, can rapidly evolve distinct life strategies that, in turn, shape both their genetic structure and their microbiomes.

## Introduction

Insects live in close association with bacteria acquired from their environment and diet. These microbial partners perform a range of functions that are essential for host survival and fitness (Gupta and Nair, 2020). The composition of these bacterial communities, collectively referred to as the microbiome, varies widely across insect taxa (Lange et al., 2023). Microbiome structure is shaped by multiple factors, including the host’s phylogenetic relationships, environmental conditions, geography, and nutritional requirements (Jackson et al., 2023; Martoni et al., 2023; Serrato-Salas and Gendrin, 2023; Yun et al., 2014). An interesting hypothesis emerging from several studies is that microbiome composition may also reflect whether the host follows a generalist or specialist ecological strategy (Brunetti et al., 2022). However, disentangling the effects of these various determinants remains challenging due to the high variability of environmental conditions.

Permanent ectoparasites that live exclusively on their hosts and feed on blood throughout their entire life cycle, such as sucking lice (Anoplura), offer a considerably simplified system for studying host–microbe interactions. Their intimate and highly specialized relationship with vertebrate host, along with strict host specificity, suggests that the host-driven factors play a major role in shaping the louse microbiome. This idea is supported by recent findings of similar bacteria in rodent fur and their lice (Říhová, 2024). As in other insects that feed exclusively on vertebrate blood, the microbiome of lice is dominated by an obligate mutualistic bacterium that supplies essential nutrients missing from the blood diet, particularly B vitamins (Duron and Gottlieb, 2020). Consequently, most studies to date have focused primarily on these obligate nutritional symbionts (Aksoy, 1995; Sasaki-Fukatsu et al., 2006; Říhová et al., 2017; Říhová et al., 2021; Říhová et al., 2022; Říhová et al., 2025; Martin Říhová et al., 2023). Only recently has the full microbiome diversity been explored in a few louse species, and even then, not in relation to the host range and spectrum (Dona et al., 2021; Deng et al., 2024).

The sucking louse *Polyplax serrata* provides an excellent model for studying the relationships between microbiome structure and its potential determinants. Although formally classified as a single species, *P. serrata* comprises a complex assemblage of lineages with distinct distributions and ecological characteristics (Štefka and Hypša, 2008; Martinů et al., 2020; Martinů et al., 2018; Martinů et al., 2025). Phylogenetic analyses suggest that this assemblage includes at least two closely related but genetically distinct species that occur across much of Europe. In both previous research and the present study, these are referred to as N (nonspecific) and S (specific) lineages. The primary distinction between them lies in host specificity: the S lineage is a strict specialist restricted to *Apodemus flavicollis*, while the more generalist N lineage can also parasitize *A. sylvaticus*. Although sucking lice are generally known for narrow host specificity, they are not always confined to a single host species, and the broader host range of the N lineage is thus not unprecedented. Many louse species are oligoxenous, parasitizing several (often closely related) host species (Durden and Musser, 1994). Moreover, host range can expand rapidly if a louse species is introduced into a new region (Wang et al., 2020). In addition to the major division between the N and S lineages, further subdivision is evident within the S lineage. Unlike the panmictic structure of the N lineage, the S lineage is divided into two geographically distinct sublineages: SW (S West) and SE (S East), which are separated by a narrow hybrid zone.

Previous comparative studies of the two major lineages have shown that the genomes of the N and S lineages are highly similar, with complete synteny interrupted only by a single small translocation (Martinů et al., 2023). In contrast, ecological differences specifically the specialist versus generalist strategies) are reflected in their different prevalences on the shared host *Apodemus flavicollis* (Martinů et al., 2025). In the present study, we build on these findings by examining the microbiome compositions of these two ecologically distinct but genetically closely related lineages. Our primary hypothesis is that the difference in host-use strategy plays a key role in shaping their microbiomes. However, we also consider and discuss additional factors that may influence microbiome structure.

## Materials and methods

### Samples and DNA preparation

Sucking lice *Polyplax serrata* were collected from *Apodemus* field mice captured in the northwest of the Czech Republic (CZ) and at several German localities (metadata for individual samples are provided in Supplementary Table S1). Permission for field work was obtained from the Committee on the Ethics of Animal Experiments of the University of South Bohemia, the Ministry of the Environment of the Czech Republic, and by the Ministry of the Agriculture of the Czech Republic (Nos. MZP/2017/630/854, 43873/2019-MZE-18134, MZP/2021/630/2459). Mice were captured using wooden snap traps. Lice were brushed from the fur and stored in 100% ethanol at −20 °C. DNA was extracted from individual lice using the Qiagen QIAamp DNA Micro Kit (Qiagen). Lineage assignment (S or N) was determined by sequencing a 379 bp fragment of the mitochondrial cytochrome oxidase subunit I gene (COI), as described in detail in Martinů et al. (2018).

### Amplicon sequencing and downstream processing

The V3–V4 region of the 16S rRNA gene was amplified from all samples using the QIAGEN Multiplex PCR Kit (Qiagen, Hilden, Germany). Amplifications included four blank (negative) PCR controls and two commercial genomic DNA (positive) controls (ATCC^®^ MSA-1000™ and MSA-1001™, each consisting of the same 10 bacterial species in different proportions). A two-step PCR protocol was applied with primers 341F and 805R containing staggered spacers and Illumina overhang adapters, followed by an index PCR to add sample-specific barcodes (Illumina 16S Metagenomic Sequencing Library Preparation Guide). Purified amplicons were quantified, pooled equimolarly, and sequenced on an Illumina MiSeq platform using v2 chemistry with 500 cycles. Demultiplexed raw reads were processed with USEARCH v11.0.667 (Edgar, 2013), including primer removal, read merging, trimming, quality filtering, and OTU clustering. Reads were merged with zero allowed mismatches, filtered using the stringent option *-fastq_maxee 1.0*, and trimmed to 400 bp. An OTU table was generated by clustering sequences at 100% identity, followed by *de novo* OTU picking with USEARCH global alignment at 97% identity, including chimera removal (Edgar, 2013). Taxonomic assignments were performed with BLASTn against the SILVA_132 database.^1^ Subsequent data filtering, rarefaction, and heatmap visualization were conducted in R using the microeco v0.16.0 package (Liu et al., 2021) and ggplot2 v3.4.2 (Ito and Murphy, 2013). Specifically, the OTU table was filtered to exclude archaeal, eukaryotic, mitochondrial, and chloroplast sequences.

### Microbiome diversity

**Figure 1:**
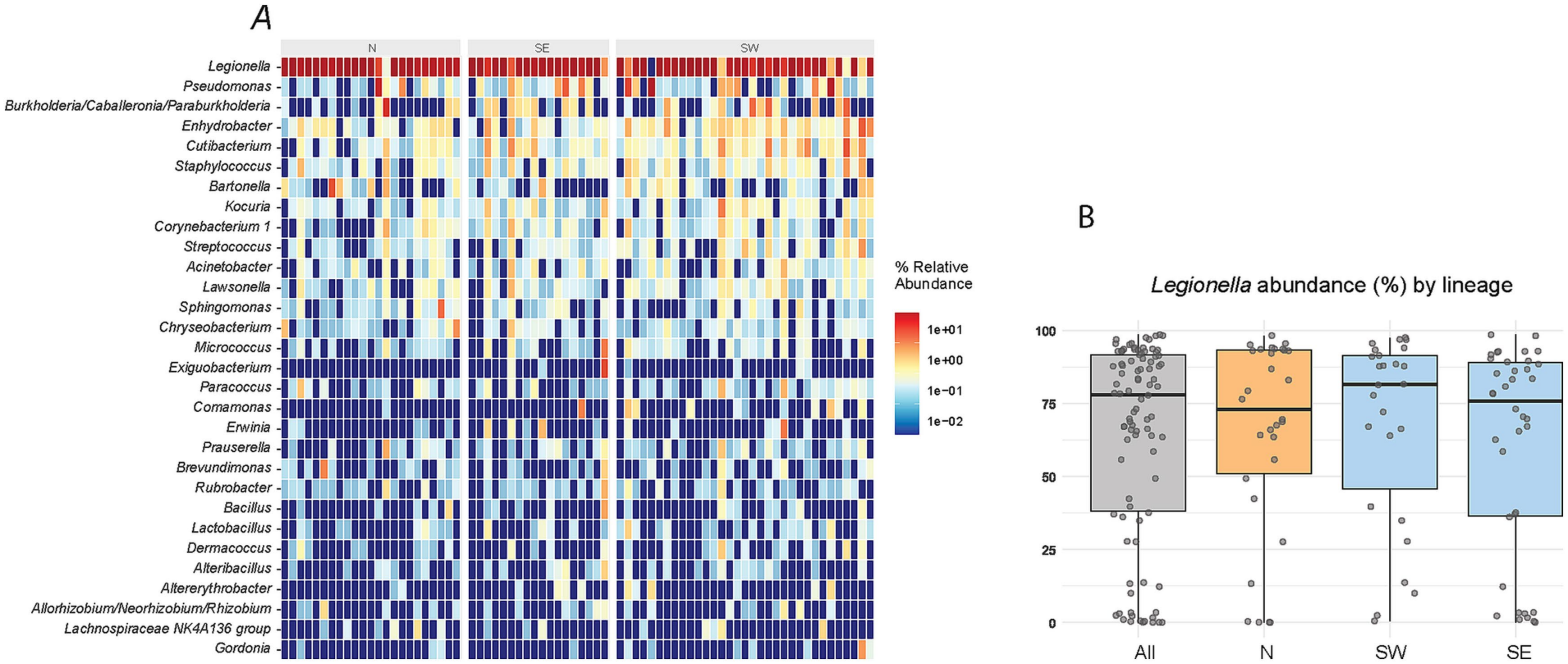
Microbiome composition based on 16S rRNA gene amplicon data. **(A)** Relative abundance of the 30 most abundant taxa. **(B)** Distribution of Legionella abundance (read counts) across samples. Boxplots show distributions for all samples, the N lineage, and the S lineage, the latter divided into two sublineages (SE, S east; SW, S west; see legend of Figure 2 for details).

**Figure 2:**
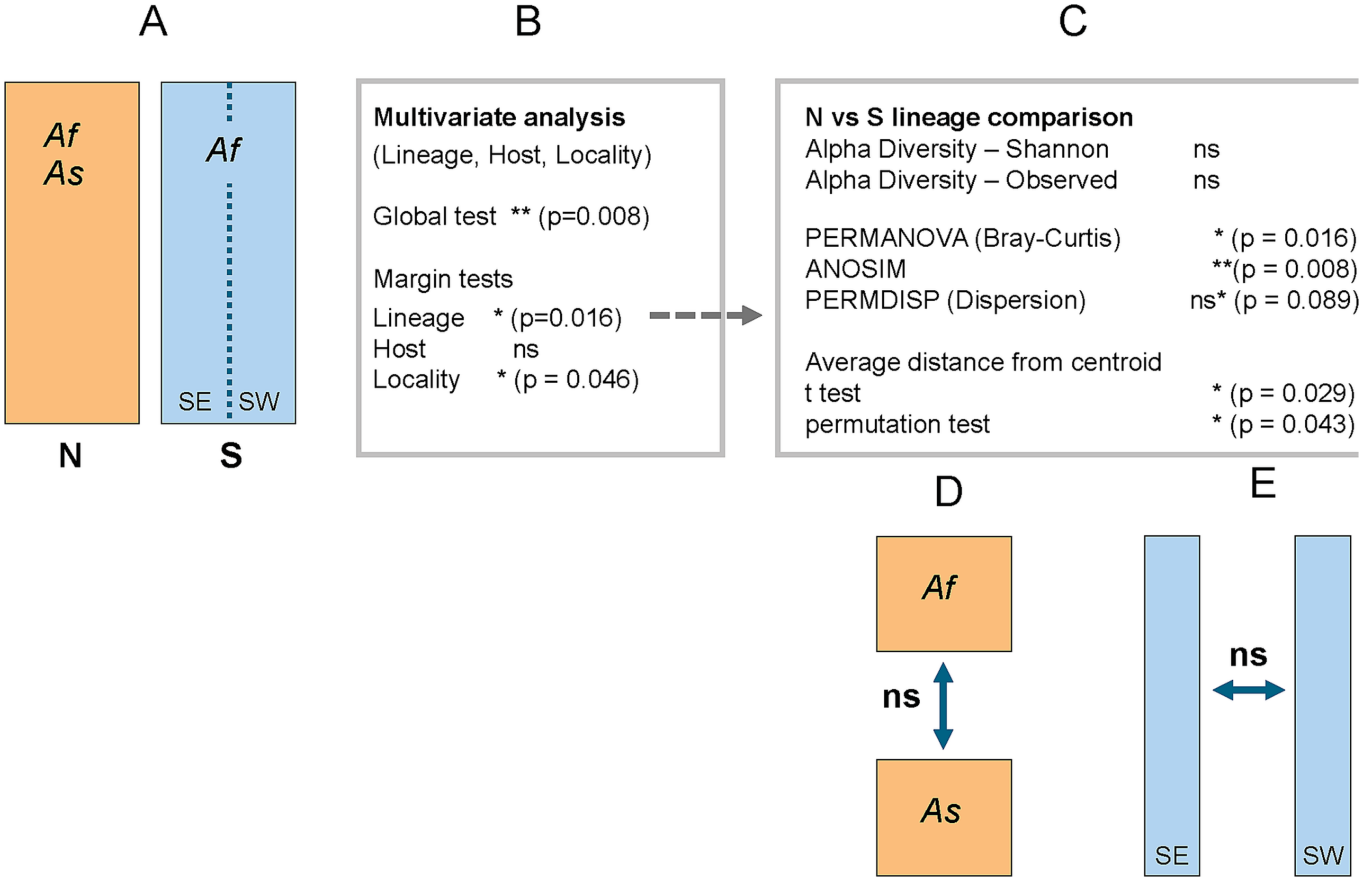
Schematic overview of statistical analyses and outcomes across the N (orange) and S (blue) lineages, sampled from two hosts: *Apodemus flavicollis* (Af) and *A. sylvaticus* (As). Significance levels are indicated as * (*p* < 0.05), ** (*p* < 0.001), and ns (*p* > 0.05); ns* denotes a non-significant but borderline result. For statistically significant results discussed in the main text, exact p-values are provided next to the corresponding symbols. **(A)** Overview of the structure of the N and S lineages. **(B)** Multivariate analysis of microbiome composition. **(C)** Follow-up comparison between N and S lineages. **(D)** Comparison of N lineage samples from two host species. **(E)** Comparison of S lineage samples from eastern and western subpopulations.

To compare microbiome structures, we applied several statistical tests across different combinations of louse lineages and sublineages (Figure 2). To minimize noise introduced by the obligate nutritional symbiont *Legionella polyplacis*, OTU1 assigned to this symbiont was excluded from the analyses. The rationale is as follows: in the majority of samples, more than 50% of reads were assigned to this OTU, with some samples reaching up to 99% (Figures 1A,B; Supplementary Table S1). On the other hand, there were also samples with low read counts for this OTU, or even complete absence. However, *L. polyplacis* is a nutritionally essential, obligate symbiont that is transovarially transmitted and fixed in *P. serrata* lice (Říhová et al., 2017). Its apparent absence in some samples likely reflects the physiological condition of the host louse rather than environmental variation. Given the high and variable abundance of *L. polyplacis* across samples, often dominating the read count, its inclusion would introduce a strong, misleading signal into the analysis. Therefore, excluding OTU1 ensures a more accurate analysis of factors underlying the microbiome structure.

For all analyses, the datasets were rarefied to 1,000 reads per sample, and samples with fewer reads were excluded (a parallel auxiliary analysis was done without rarefication; see Discussion). The main comparison was conducted between the N and S lineages. All analyses were performed in the R environment (R Core Team, 2014), including data cleaning, statistical testing, and result visualization. The analysis utilized the packages microeco, vegan (Oksanen et al., 2025), and ggplot2 v3.4.0. To compare microbial diversity, we first conducted multivariate analyses to assess the combined and individual effects of the variables Lineage, Host, and Locality. Based on these results, we further analyzed the effect of Lineage on alpha and beta diversity using several complementary methods. Alpha diversity was compared using the Shannon index. Beta diversity was assessed from multiple perspectives. A basic comparison of beta diversity was performed using PERMANOVA based on Bray–Curtis distances. To evaluate differences in sample distances to group centroids, we applied two approaches: PERMDISP was used to assess dispersion (i.e., variability in distances of individual samples to the group centroid), while mean distances to group centroid were compared using t-test and a permutation test. To control for the possible effect of the within-lineage characteristics, we further compared the N lineage samples from the two different hosts (*A. flavicollis* and *A. sylvaticus*), and S samples from the two mitochondrial lineages (SW and SE). Additionally, we assessed the possible influence of sex and developmental stage by comparing microbiome beta diversity among females, males, and nymphs. To evaluate whether the distribution of sexes and developmental stages differed between the S and N lineages, we used the chisq.test() function in R.

### Relation between population structure and host specificity

To determine whether the N lineage is entirely “generalistic” or instead forms host-determined clusters, we analyzed the population structure of the lice using two complementary approaches: mitochondrial DNA-based phylogeny and genome-wide nuclear SNP-based genetic structure. For this purpose, 38 lice from both host species (*A. sylvaticus* and *A. flavicollis*) sampled from the same localities where possible, were selected and their genomes re-sequenced. DNA concentrations were verified using a Qubit 2.0 Fluorometer (Invitrogen) with high-sensitivity reagents. Ultralow Input Libraries (Tecan) were prepared, and 150-bp paired-end sequencing was carried on one S4 Illumina Novaseq 6,000 flow cell at the W. M. Keck Center (University of Illinois, Urbana, IL, United States).

For SNP analysis, genomic reads were mapped to the reference genome of *Polyplax serrata* S lineage (GCA_037055385.1) using bowtie2 (Langmead and Salzberg, 2012). The reference was indexed using SAMtools (Li et al., 2009), and a sequence dictionary was created with CreateSequenceDictionary in Picard version 2.0.1.^2^ SAM files were converted to sorted BAM files and indexed with SAMtools. Duplicated sequences were removed using Picard 2.0.1 and mapping success was assessed with qualimap.^3^ Variant calling was performed with GATK following the “Best Practices” workflow (Van der Auwera et al., 2013), and the resulting SNP set was filtered using the thresholds QD < 2.0, FS > 60.0, MQ < 40.0, and MQRankSum < −12.5. In the R package SNPRelate,^4^ SNPs with a minor allele frequency (MAF) < 0.05 and linkage disequilibrium (LD) > 0.2 were removed. Population structure was reconstructed using PCA within the same package.

Like in other *Polyplax* lice, the mitochondrial DNA of *P. serrata* is organized into 11 separate minichromosomes (Martinů et al., 2020). To recover these minichromosomes from N lineage lice, we performed metagenomic assemblies using SPAdes (Bankevich et al., 2012). For each assembly, we created a database using the algorithm implemented in Genious (Kearse et al., 2012). Within each database, contigs corresponding to minichromosomes were identified via BLAST, using previously published minichromosomes from the S lineage of *P. serrata* as queries (Martinů et al., 2020). Minichromosomes were successfully extracted from 32 of the 38 analyzed samples. These sequences were aligned using a codon-based algorithm implemented in Geneious, and the resulting alignments were concatenated into a single nucleotide matrix. Phylogenetic reconstruction was performed in IQ-TREE (Trifinopoulos et al., 2016), treating each minichromosome as partition for which IQ-TREE was used to determine the best-fitting model for each partition (Supplementary Data).

### Comparison of genetic diversities of the lice and the microbiomes

To evaluate possible effect of louse genetic diversification on microbiome beta diversity, we performed two complementary tests. First, nucleotide diversity (*π*) was calculated and compared between the two louse lineages. For this purpose, we used SNP datasets derived from specimens with matching microbiome profiles. For the N lineage, we selected 28 of the specimens that had been re-sequenced as described above. For S lineage, 64 specimens were sequenced on the same Illumina NovaSeq lane, and SNP datasets were generated using the same pipeline. Nucleotide diversity was then calculated separately for each lineage from the VCF file using the vcfR package in R (Knaus and Grünwald, 2017). Second we assessed the correlation between louse genetic diversification and microbiome beta diversity. Raw genotype data in VCF format were first converted into PLINK binary files (.bed, .bim, .fam), and genotypes were exported into a numeric matrix using PLINK’s—recode A option (Purcell et al., 2007). Pairwise genetic distances between individuals were calculated in Python (using the Euclidean distance metric) with the pdist and squareform functions from the SciPy library (Virtanen et al., 2020). The resulting genetic matrices for the N and S lineages were compared to the Bray-Curtis distance matrices of their microbiomes using a Mantel test implemented in R with vegan package. Prior to testing, both matrices were reordered to ensure that samples were matched. The Mantel test was performed with 999 permutations using Pearson correlation to assess the relationship between genetic and microbiome dissimilarities. To visualize this relationship, the lower-triangular elements of both matrices were extracted and plotted against each other using ggplot2, with a regression line overlay and the Mantel correlation coefficient (r) and associated *p*-value displayed in the plot title.

## Results and discussion

### Microbiome richness and composition

The amplicon screening identified average of 68 distinct OTUs per sample, showing that despite their limited access to diverse environments, the permanent blood-feeding ectoparasites *P. serrata* harbor a relatively rich microbiome. This estimate is based on samples that retained sufficient sequencing depth and were included in the analysis following rarefaction (Figure 1A; Supplementary Table S1). Placing this number in the context of other exclusive blood feeders is difficult due to scarcity of comparable studies. Moreover, evaluating microbiome richness in these groups is further complicated by the dominance of one or few symbionts. For example, in tsetse flies, 99,7% of amplicon reads were assigned to the three known symbionts *Wigglesworthia*, *Sodalis*, and *Wolbachia* (Gaithuma et al., 2020). Similarly, in the bedbug *Cimex hemipterus*, the two most abundant symbionts, *Wolbachia* and *Symbiopectobacterium*, accounted for up to 99% of the reads (Lim and Ab Majid, 2021). In our data, the most abundant OTU1 was assigned to the genus *Legionella*, clearly corresponding to the obligate symbiont *L. polyplacis* (Říhová et al., 2017). It was detected in the majority of the samples; however, its read abundance varied substantially, ranging from nearly 100% to near or complete absence in a few samples (Figure 1B; Supplementary Table S1).

Such variation in the proportion of reads assigned to an obligate symbiont across individual hosts (with some samples even lacking detectable symbiont reads) has also been reported in human lice *Pediculus humanus* (Agany et al., 2020). Because obligate symbionts are essential for their hosts, these differences likely reflect the physiological or developmental stage of the host, rather than factors driving microbiome variation examined in our study. This interpretation is supported by the read distribution across our samples (Figure 1B), which is continuous between 0 and 100%, rather than binary (present/absent). As explained in Methods, OTU1 was excluded from subsequent analyses to avoid introducing of misleading signal.

### Microbiome diversities

The global PERMANOVA model, incorporating Lineage, Host, and Region as explanatory variables (see Figure 2 for the complete statistical workflow), explained approximately 14% of the total variation in microbiome structure (*p* = 0.008, *R*^2^ = 0.14, *F* = 1.26). This indicates a moderate but statistically significant combined effect of the three predictors. Marginal tests identified Lineage (N versus S) as the primary driver of variation (*p* = 0.016), while Region (*p* = 0.046) showed a borderline significant effect, and Host (*p* = 0.178) did not reach statistical significance. Consequently, follow-up analyses focused on comparing diversity measures between the N and S lineage microbiomes.

**Figure 3:**
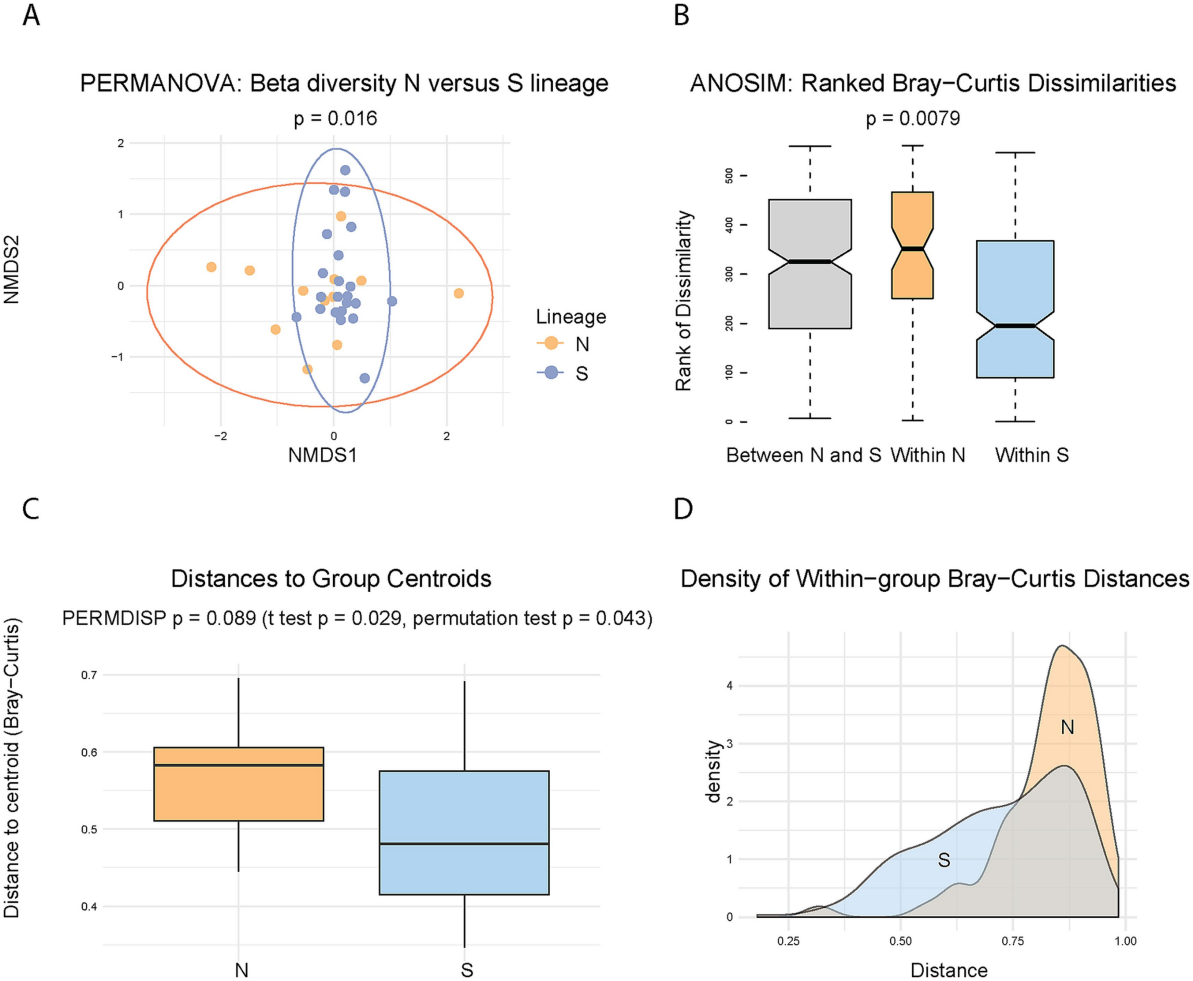
Comparison of microbiome diversity between N and S lineages. **(A)** Ordination plot of beta diversity based on Bray–Curtis distances, with 95% confidence ellipses for each lineage. PERMANOVA analysis indicates a significant difference in microbiome composition between lineages (*p* = 0.016). **(B)** ANOSIM boxplot showing ranked dissimilarities within and between N and S lineages, supporting significant compositional differences (*R* = 0.214, *p* = 0.0079). **(C)** Boxplot of sample distances to the group centroid. Significance values are shown for the tests: PERMDISP assessing variability (dispersion) of the distances (*p* = 0.089), and two tests comparing average distances to the centroid. **(D)** Density plot of distances to centroid, visualizing the same data as in **(C)** and highlighting differences in distribution shape between lineages.

These analyses revealed that the two lineages exhibit similar alpha diversity, as measured by the Shannon index (*p* = 0.70) (Supplementary Figure S1A), indicating no significant difference in overall richness and evenness of their microbiomes. In contrast, microbiome composition differed significantly between lineages, as supported by both PERMANOVA (Bray–Curtis, *p* = 0.016, R^2^ = 0.06, *F* = 1.96) (Figure 3A) and ANOSIM (*R* = 0.214, *p* = 0.0079) (Figure 3B). Visual inspection of the ordination plots suggests that the observed differences may be influenced by greater heterogeneity within the N lineage microbiomes (Figures 3A,B). This apparent heterogeneity could stem from two distinct, though related, sources: increased *dispersion* (greater variability in distances of individual samples from the group centroid), and/or a higher *average* distance from the centroid, regardless of within-group variability. To test these possibilities, we first assessed group dispersion using PERMDISP, which did not reach the conventional significance threshold (*p* = 0.089) (Figures 3C,D), although the result approached significance and may indicate a trend. In contrast, both a *t*-test and a permutation test comparing the mean distances to centroid between lineages yielded significant results (*p* = 0.029 and *p* = 0.043, respectively) (Figure 3C). These findings suggest that while the difference in dispersion is not statistically conclusive, the N lineage tends to harbor microbiomes that are, on average, more dissimilar from the group centroid. Thus, while the difference in microbiome composition between lineages is clearly supported, the extent to which this difference reflects increased heterogeneity within the N lineage remains suggestive rather than definitive.

As discussed above, investigating microbiomes in obligate blood-feeders is complicated by the dominance of obligate nutritional symbionts. In our study, all analyses were performed after excluding *L. polyplacis* (see also Materials and Methods), which resulted in a substantial reduction in data. This necessitates careful consideration of statistical power in subsequent analyses. The key finding that the N and S lineages differ in microbiome beta diversity is based on the test with statistical power ≈ 0.39, which is considerably below the commonly recommended threshold of 0.80. Nevertheless, several lines of evidence support the validity of the observed pattern. First, low statistical power is primarily a concern because it increases the risk of failing to detect true effects (Type II error). However, in this case, we are evaluating the reliability of an effect that was detected. This finding is supported by a statistically significant *p*-values of PERMANOVA analysis (*p* = 0.016) and even higher significance yielded by ANOSIM (*p* = 0.0079). Therefore, despite the low power, the robustness of the observed signal suggests that the detected difference in microbiome beta diversity between the lineages is unlikely to be a false positive. Second, when calculated using Jaccard rather than Bray-Curtis distances, PERMANOVA analysis yielded significant result with an even lower *p*-value (0.009). Finally, an auxiliary analysis using non-rarefied data (which retained a larger number of samples) yielded similar results (see Supplementary Table S2). This further supports the consistency of the main signal, even though the non-rarefied dataset may be subject to additional sources of bias or artifacts.

Numerous factors can influence differences in microbiome structure across species or populations, including geography, trophic status, dietary source, and environment. Among these, geography was explicitly tested in our multivariate model and showed borderline statistical significance (*p* = 0.046). Its effect was notably weaker compared to that of Lineage. This corresponds well with the geographical structure of our sampling. Most samples come from several localities in Bohemia, all of the localities contain both lineages. There are two exceptions: the populations from Bavaria and Saxony contain only N lineage lice. However, when these samples were removed from the analysis, the difference between the N and S remained significant (Supplementary Figure S1B).

Another potential factor influencing microbiome composition is the trophic status of the insect host. In the blood-feeding species *Cimex hemipterus*, it has been shown that microbiome composition varies with trophic state: starved individuals possess a richer microbiome than blood-fed ones. However, trophic status can very likely be excluded as an explanation for our results. A crucial difference exists between the feeding strategies of bed bugs and sucking lice. Bed bugs feed only once every few days (Reinhardt and Siva-Jothy, 2007), experiencing a cyclical shift between fed and starved states. In contrast, sucking lice feed multiple times per day (Schaub et al., 2012), resulting in a more stable trophic state. More importantly, since the only significant predictor of microbiome variation identified in our analysis was the division between the N and S lineages, it is unlikely that trophic status would follow this same pattern. As explained above, trophic status is likely reflected in the abundance of the obligate symbiont *L. polyplacis*, which was excluded from the analysis for this very reason. Apart from the trophic status, the sex or developmental stage could theoretically influence microbiome structure. Given the low prevalence of the lice, we sampled both sexes, and from some host individuals, only nymphs were available (see metadata in Supplementary Table S1). However, two lines of evidence suggest that this parameter did not significantly affect the observed differences. First, comparison of microbiome beta diversity among females, males, and nymphs revealed no significant differences (PERMANOVA: *p* = 0.329; ANOSIM: *p* = 0.946). Second, the distribution of the sexes and developmental stages did not differ significantly between the S and N lineages (Pearson’s Chi-squared test: *p* = 0.307).

**Figure 4:**
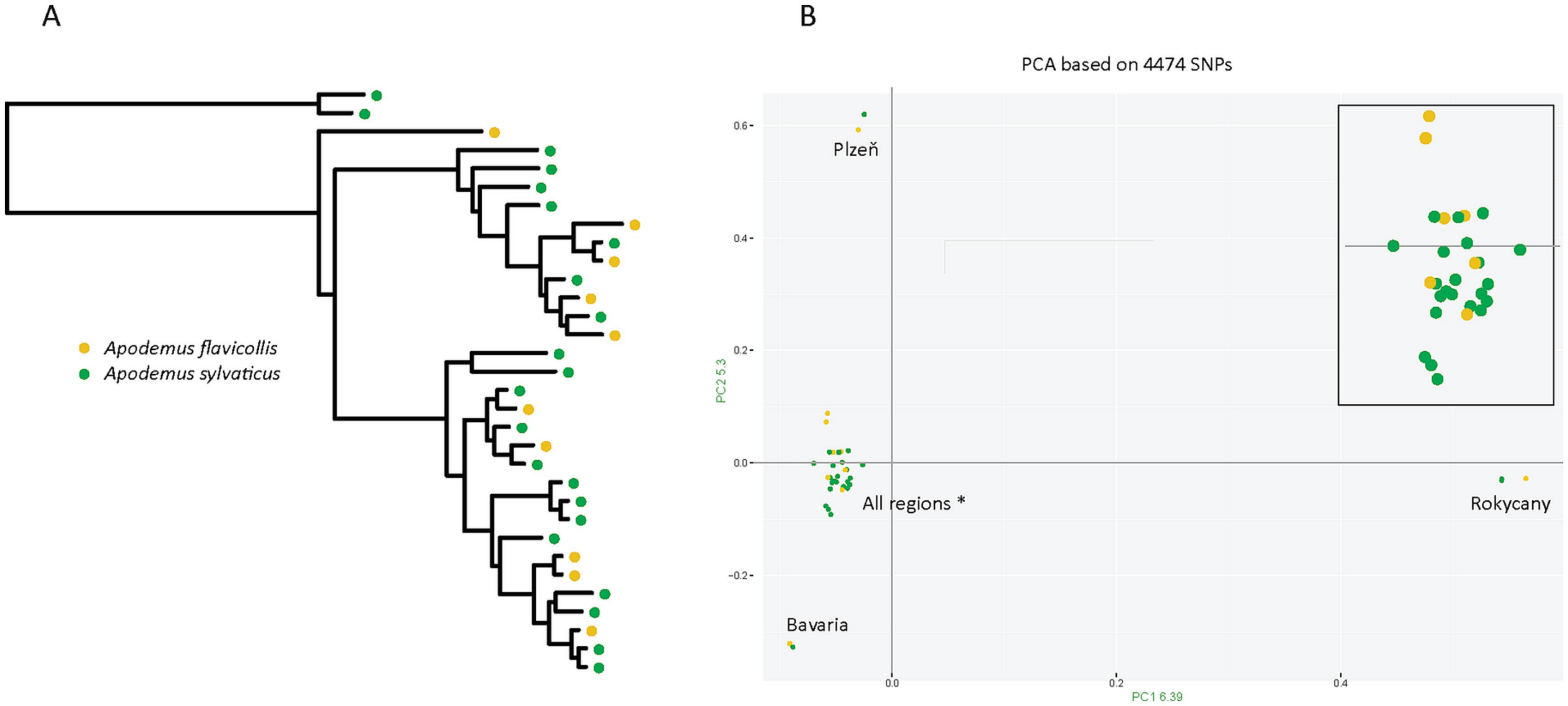
Rodent host distribution mapped onto the genetic structure of the N lineage. **(A)** Maximum-likelihood phylogenetic tree inferred with IQ-TREE from a concatenated alignment of eight mitochondrial minichromosomes (10,754 positions; partitions and substitution models listed in Supplementary Data). **(B)** Principal Component Analysis (PCA) based on SNP data. Both host species are intermixed across all four genetic clusters (Plzeň, Rokycany, Bavaria, and a large cluster labeled “All regions*” that includes individuals from all sampled sites). For clarity, a zoomed-in view of the large cluster is shown in the upper right corner.

The most likely explanation for the observed microbiome differences is ecological variation, specifically host specificity. The S lineage is restricted to a single rodent host, *Apodemus flavicollis*, while the N lineage parasitizes two rodent species, *A. flavicollis* and *A. sylvaticus*. Mapping rodent hosts on genetic structure of the N lineage confirms that these lice frequently switch from one host species to another, rather than forming host specific clusters (see details below) (Figure 4A). This difference in host usage may influence microbiome composition in two ways. First, microbiome composition may reflect the *current host*, meaning it can change rapidly, in each louse individual being determined by the host on which the louse is presently residing. Second, in a longer ecological time, host switching creates a more dynamic environment, leading to more diverse microbiomes. This factor we term as the *host-usage strategy*. A targeted comparison of N lineage lice from the two host species revealed no significant difference in microbiome composition. Samples from *A. flavicollis* and *A. sylvaticus* were intermixed in ordination plots, and statistical analysis yielded non-significant results (*p* = 0.28) (Supplementary Figure S1C). These findings do not support the *current host* hypothesis.

The *host-usage strategy*, specifically, the broader spectrum of rodent hosts exploited by the N lineage, offers the most plausible explanation for the observed microbiome differences. From a biological perspective, frequent switching between rodent host likely exposes N lineage lice to more complex and variable environmental conditions, which in turn promotes greater microbiome diversity over time. Due to these different strategies and distinct evolutionary histories, the S and N lineages also exhibit divergent genetic structures. Based on single nucleotide polymorphism (SNP) data, the N lineage displays significantly higher nucleotide diversity than the S lineage (0.051 vs. 0.006). This finding is consistent with Nadler’s hypothesis (Nadler, 1995) or a similar Specialist-Generalist Variation Hypothesis (Li et al., 2014), which posit that generalists, owing to more frequent opportunities for dispersal, tend to maintain populations with higher local diversity (Li et al., 2014). This raises the key question of whether microbiome diversity differences are driven directly by the *host-usage strategy* (as the ultimate factor shaping ecological exposure) or indirectly through the distinct genetic structures of the N and S lineages (which themselves are consequences of the *host-usage strategies*). To address this, we compared pairwise Bray-Curtis distances derived from microbiomes with Euclidean distances derived from SNPs. The Mantel test revealed very weak correlations, with borderline nonsignificance (N lineage: *r* = 0.20, *p* = 0.61; S lineage: *r* = 0.07, *p* = 0.055). Based on these results and the overall biology of the system, we propose that the direct effect of *host-usage strategies* provides the more plausible explanation for the microbiome diversity differences.

### Host independent structure of the N lineage

The interpretation provided above assumes a real “generalistic” nature of the N lineage, that is, frequent random switches between the two *Apodemus* species (as contrast to few host specific clusters). This assumption has been confirmed by both phylogeny based on mitochondrial genome and population structure derived from genome-wide SNPs (Figure 4B). In the phylogenetic tree, lice from different hosts are interspersed across the topology with only occasional monophyly of samples from the same host. Similarly, in PCA, most cluster without host-specific division. A few samples were positioned far from the main cluster by large distances on both axes, but each of these small outlier groups contained samples from both host species.

## Conclusion

Our analyses suggest that in *P. serrata* lice, the specialist versus “generalist” strategy (i.e., exploiting a single versus two host species) influences microbiome diversity, with the “generalist” lineage harboring more heterogeneous microbiomes. This result fits into a broader pattern observed across insect studies, where similar ecological factors play an important role in shaping the evolution of microbiome diversity. For example, in Chrysomelidae beetles, generalist species harbor more diverse microbiomes than specialists (Brunetti et al., 2022). Within Anoplura, significant difference was detected in human louse *Pediculus humanus* between the head and body ecotypes, even though these ecotypes do not form mutually exclusive monophyletic clusters (Agany et al., 2020). With respect to our model species *P. serrata*, the findings reported here extend the previous observations on ecological differences between the N and S lineages. Taken together, the accumulated results illustrate how closely related cryptic species with highly similar genomes, living in sympatry in the same environment, can rapidly evolve different life strategies that, in turn, shape both their genetic structure and the composition of their microbiomes.

## Data Availability

The data presented in this study are publicly available in the NCBI repository (https://www.ncbi.nlm.nih.gov/). Sequencing data are available under accession PRJNA1335907; metagenomic data under accessions SAMN52029611-SAMN52029693 and SAMN52353852-SAMN52353859; and amplicon sequencing data under accessions SAMN52070577-SAMN52070662.

## Footnotes

^1^ https://www.arb-silva.de/fileadmin/silva_databases/release_132/Exports/SILVA_132_SSURef_tax_silva_trunc.fasta.gz

^2^ https://broadinstitute.github.io/picard/

^3^ http://qualimap.bioinfo.cipf.es/

^4^ https://doi.org/10.18129/B9.bioc.SNPRelate
