# Supplementary material for: Generalist vs. specialist strategy shapes microbiomes in blood feeding parasite Polyplax serrata
Source: Front Microbiol. 2025 Nov 21;16:1720127. doi: 10.3389/fmicb.2025.1720127 (PMC12679383; doi:10.3389/fmicb.2025.1720127)
Supplement: Supplementary file 1 [file Data_Sheet_1.pdf]

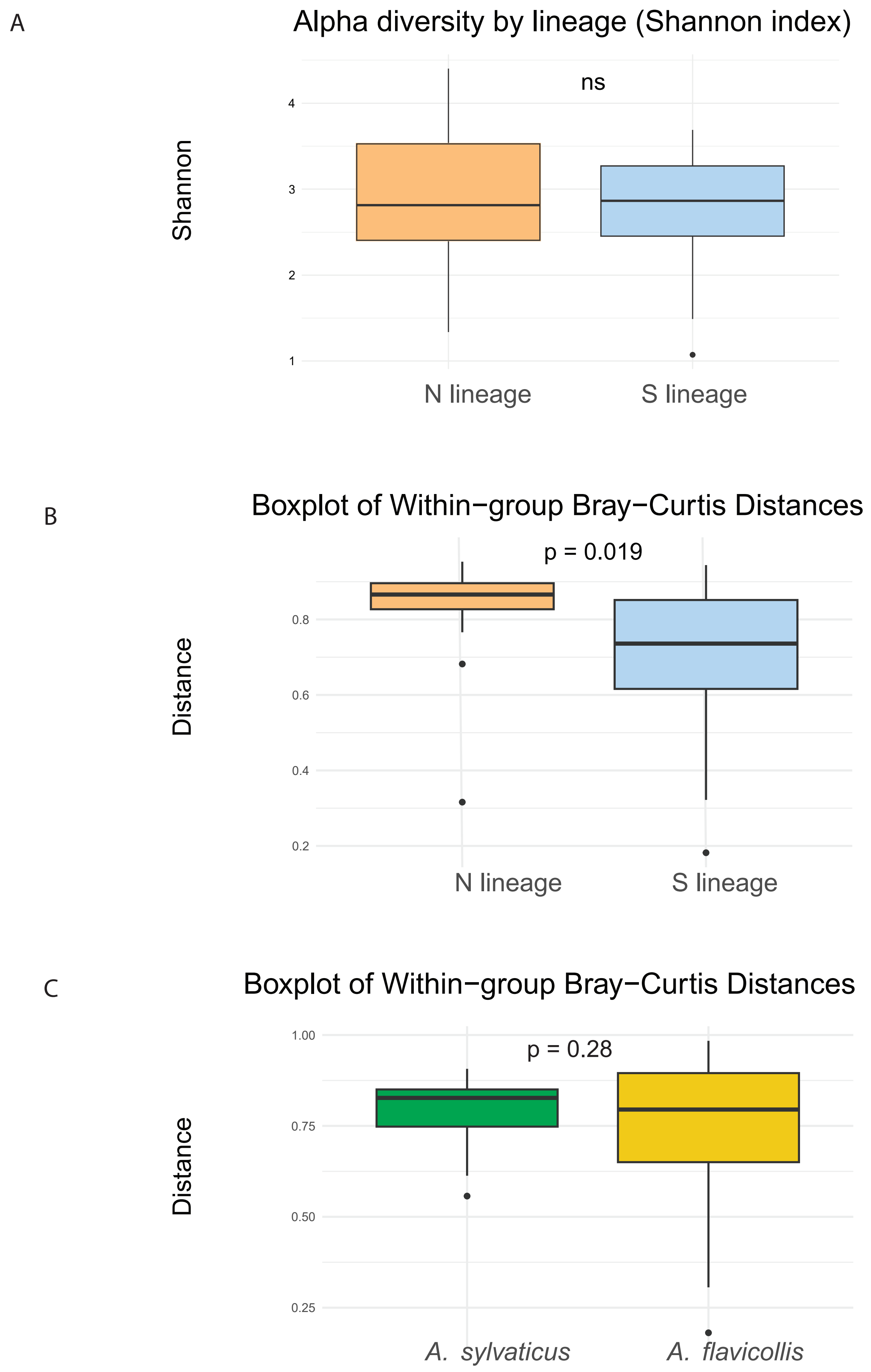

Supplementary figure S1. (A) Boxplots of alpha diversity (Shannon index). (B) PERMANOVA analysis showing group separation after removal of Bavarian and Saxony samples. (C) PERMANOVA results comparing samples from two host species.
